# Variation in *KRAS* driver substitution distributions between tumor types is determined by both mutation and natural selection

**DOI:** 10.1038/srep21927

**Published:** 2016-02-23

**Authors:** Sheli L. Ostrow, Einav Simon, Elad Prinz, Tova Bick, Talia Shentzer, Sima S. Nagawkar, Edmond Sabo, Ofer Ben-Izhak, Ruth Hershberg, Dov Hershkovitz

**Affiliations:** 1 TICC, Technion Integrative Cancer Center at the Ruth and Bruce Rappaport Faculty of Medicine, Technion-Israel Institute of Technology, Haifa, Israel; 2 Department of Genetics and Developmental Biology, the Ruth and Bruce Rappaport Faculty of Medicine, Technion-Israel Institute of Technology, Haifa, Israel; 3 Department of Pathology, Rambam Health Care Campus, Haifa, Israel; 4 Institute of Oncology, Rambam Health Care Campus, Haifa, Israel

## Abstract

Different tumor types vary greatly in their distribution of driver substitutions. Here, we analyzed how mutation and natural selection contribute to differences in the distribution of *KRAS* driver substitutions between lung, colon and pancreatic adenocarcinomas. We were able to demonstrate that both differences in mutation and differences in selection drive variation in the distribution of *KRAS* driver substitutions between tumor types. By accounting for the effects of mutation on the distribution of *KRAS* driver substitutions, we could identify specific *KRAS* driver substitutions that are more favored by selection in specific tumor types. Such driver substitutions likely improve fitness most when they occur within the context of the tumor type in which they are preferentially favored. Fitting with this, we found that driver substitutions that are more favored by natural selection in a specific type of tumor tend to associate with worse clinical outcomes specifically in that type of tumor.

The identity of which driver substitutions will appear within a given tumor is determined by both somatic mutation patterns and by natural selection^1^,^2^,^3^,^4^,^5^. Different tumors may substantially vary in the biases of their mutational process, as a function of their type (e.g. lung vs colon) as well as a function of exposure to carcinogens (e.g. smokers vs. non-smokers)^2^. Such differences could lead to differences in the frequency of occurrence of specific drivers and therefore influence the identity of the drivers that are eventually present within a tumor. At the same time different driver substitutions may differ in their functional effects. Given their different functional effects it is quite likely that natural selection occurring within a tumor will not influence various driver substitutions in the same manner. Since the cellular microenvironment of various tumors can also be quite different, it is also very likely that between tumor types and depending on exposure to carcinogens, selection could also affect the same drivers differently.

Following the occurrence of a mutation, natural selection can affect it in three ways: (1) If the mutation has no effect on function, and therefore no effect on fitness, selection will simply not affect its fate. (2) If the mutation increases cellular fitness (is adaptive), positive natural selection will increase the likelihood it will increase in frequency. (3) If the mutation decreases cellular fitness (is deleterious) purifying natural selection will increase the likelihood that the mutation is removed from the cellular population. Those mutations that are allowed to persist within the population and which we can see when sequencing genes or genomes are referred to as substitutions. As discussed above, the identity of the substitutions we observe will depend on both the biases of mutation (that may lead to certain mutation types occurring more frequently than others^6^) and the action of natural selection.

In this study we focused on determining which driver substitutions within the *KRAS* oncogenic gene are most and least favored by selection in different types of tumors.

The *KRAS* gene from the ras family encodes a small GTPase protein^7^. Substitutions in *KRAS* are the most common activating substitutions in cancer and mostly involve codons 12, 13 and 61 of the gene^8^. These substitutions are associated with reduced survival in non-small cell lung cancer^9^ and colon carcinoma^10^. *KRAS* driver substitutions also predict response to targeted anti-cancer treatment^11^.

It was already noted more than two decades ago that different tumors have different *KRAS* driver substitution subtypes. For example, colon carcinoma shows more G > A transitions while in pancreatic carcinoma the more common *KRAS* substitutions are G > T or G > C transitions^12^. The differences in *KRAS* substitution subtypes in different cancers might be entirely explained by differences in mutation patterns between the cancer types. Such differences in mutation may be due to variation in exposure to carcinogens and/or to differences in the expression of DNA repair proteins between tissues and/or tumor types. Differences in mutational biases between tumor types and as a function of smoking carcinogen exposure have been well documented^2^. Alternatively, some of the differences in the distribution of *KRAS* substitutions might be attributed to differences in natural selection, due to differences in *KRAS* protein function within the different tumor types. Indeed it has been shown that *KRAS* substitutions can differ in their downstream effects. For example, the c.35G > A (p.G12D) substitution is associated with PI3K and MEK activation whereas c.34G > T (p.G12C) and c.35G > T (p.G12V) induce Ral-signaling and downregulation of AKT^13^. These substitution specific downstream effects might explain the differences between the substitutions with regard to prognosis^13^,^14^,^15^ and treatment response^16^,^17^,^18^,^19^ and have led to the development of anti-cancer treatments that target specific *KRAS*-substitution-subtypes^20^.

In this study we demonstrate that some of the variation in the distribution of *KRAS* substitutions between tumor types is explained by differences in mutational biases. However, much of this variation cannot be explained by differences in mutation, and is rather the result of differences in the identity of the substitutions most favored by natural selection. Finally, we found that there tends to be an association between the presence of *KRAS* substitutions that are favored within specific tumor types and clinical outcomes.

## Results

### Cancer types vary in their distribution of *KRAS* driver substitutions

Three tumor types were analyzed in this study: lung adenocarcinoma (LUAD), colon adenocarcinoma (COAD) and pancreatic adenocarcinoma (PAAD). Two datasets were used to analyze the distribution of *KRAS* codon 12 and 13 driver substitutions within these tumor types. The datasets used were the Cancer Genome Atlas (TCGA)^21^ (Table S1), and a dataset we collected ourselves locally of three cohorts of cancer patients. These cohorts included 346 cases of LUAD, 314 cases of COAD and 47 cases of PAAD.

**Figure 1:**
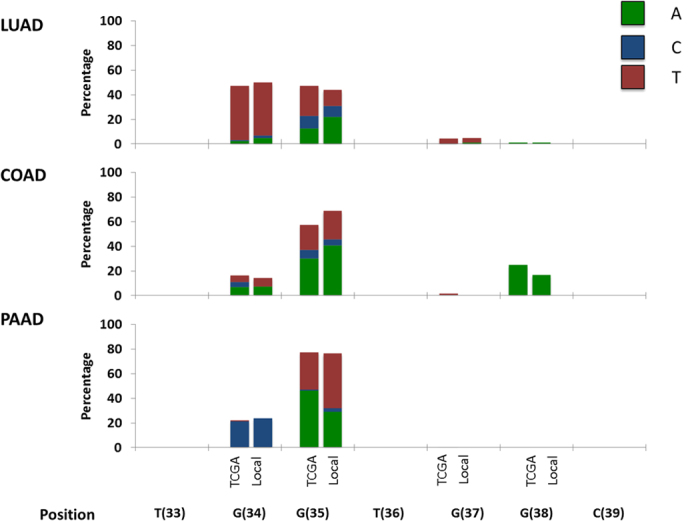
Observed distributions of *KRAS* codon 12 and 13 driver substitutions vary greatly between studied tumor types. At the same time, these distributions are highly congruent between the publically available Cancer Genome Atlas (TCGA) dataset and our own local patient cohorts. Identities of substitutions are indicated by nucleic acid name and position (brackets) in the *KRAS* gene’s cDNA, at the bottom of the figure. LUAD = lung adenocarcinoma, COAD = colon adenocarcinoma, PAAD = pancreatic adenocarcinoma.

As expected from previous studies^22^, the frequency with which tumors harbored *KRAS* driver substitutions varied between tumor types, and were much higher for PAAD compared to LUAD and COAD (Table 1). From the cases in which a tumor carried a *KRAS* substitution we could calculate the relative frequencies at which each of the twelve possible codon 12 and 13 *KRAS* driver substitutions occurred within the different tumor types. The relative frequencies of the different possible *KRAS* substitutions within each tumor type were highly similar between the local cohort, and the TCGA, and showed no significant differences between the two datasets (*P* > 0.05 for all comparisons according to a χ^2^ test, Fig. 1, Table S2). At the same time, the three tumor types showed a significantly different distribution of *KRAS* substitutions (*P* ≪ 0.001 for all comparisons, Fig. 1, Table S2). While for lung samples the c.34G > T substitution was the most common, in the remaining two cancer types the most common *KRAS* driver substitution was c.35G > A. Additionally, according to the two datasets, c.34G > C was present in 20–24% of pancreatic adenocarcinoma cases and was nearly absent in the other tumors, whereas, c.38G > A, the third most common substitution in colon carcinoma (17–25% of cases, in the two different datasets) was very rarely identified in the other tumors.

### Tumor types differ in their patterns of mutation

Differences in the distribution of *KRAS* driver substitutions could be the result of both variation in mutational biases and differential selection acting on the various *KRAS* driver substitutions. To determine the contribution of mutational biases differences in generating the observed variation in *KRAS* driver substitution distribution, we wanted to characterize the mutational biases of the three studied tumor types. In order to estimate the mutational biases of each tumor type we used data from the TCGA. The TCGA contains data of full exome sequencing of tumors and paired healthy tissues from a large number of patients suffering from the three types of cancer studied here (Table S1)^21^. From these data we could extract synonymous somatic substitutions, defined as somatic substitutions that alter the nucleotide sequence of a protein-coding gene, but do not alter its amino acid sequence. Such synonymous substitutions are less likely to be affected by selection than non-synonymous substitutions that do alter amino acid sequences. To further minimize the involvement of selection we focused on synonymous substitutions that are found only within a single patient. As a group, such non-reoccurring synonymous substitutions (NRSSs) should be less affected by natural selection, compared to those substitutions that appear in many different patients. Biases in the patterns of such NRSSs therefore likely better reflect the biases of the mutational process occurring within each tumor type.

**Figure 2:**
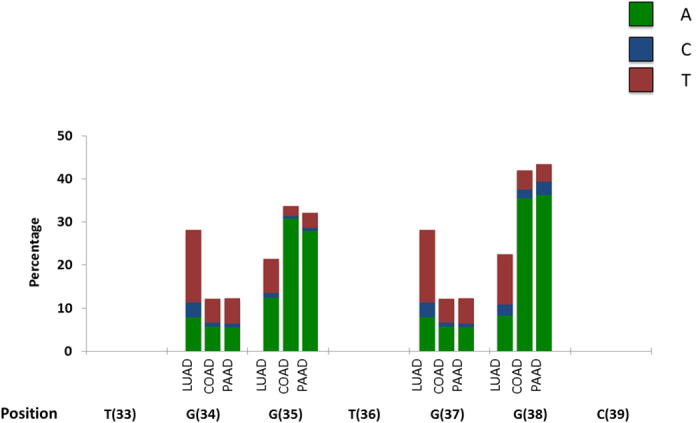
Expected relative frequencies of the different *KRAS* codon 12 and 13 driver mutations vary greatly between tumor types. In order to estimate the expected distribution of *KRAS* codons 12 and 13 substitutions, under a model in which the relative frequencies of each substitution is determined solely by mutation, we used data of synonymous non-reoccurring mutations from the TCGA. Analysis also accounted for the sequence context of each possible nucleotide change, as detailed in the methods section. Identities of mutations are indicated by nucleic acid name and position (brackets) in the *KRAS* gene’s cDNA, at the bottom of the figure. LUAD = lung adenocarcinoma, COAD = colon adenocarcinoma, PAAD = pancreatic adenocarcinoma.

All 12 known driver substitutions within *KRAS* codons 12 and 13 occur within four nucleotide positions and involve a change of a guanine nucleotide to another nucleotide (G > N). We therefore estimated the expected frequency with which different G > N mutations occur based on our data of G > N NRSSs. Mutation rates are known to be context dependent (i.e. affected by the identity of flanking nucleotides). The four positions within *KRAS* codons 12 and 13 that can harbor driver substitutions appear in the following contexts: tGg, gGt, tGg, and gGc, where the G in which the driver substitution can occur appears capitalized and the flanking positions appear in lower case. Using the data of NRSSs, we performed a context corrected calculation for the expected relative frequency of each G > N mutation (see Materials and methods). We found that the relative frequency with which mutations were expected to occur within the four G nucleotides of codons 12 and 13 differed significantly between the three examined tumor types (Fig. 2, Table S3, *P* < 0.005 for all comparisons, using a χ^2^ test). Thus, differences in mutational biases have the potential to contribute to the observed variation found between tumor types in the distribution of *KRAS* driver substitutions.

### Natural selection sometimes favors different *KRAS* driver substitutions in different tumor types

**Figure 3:**
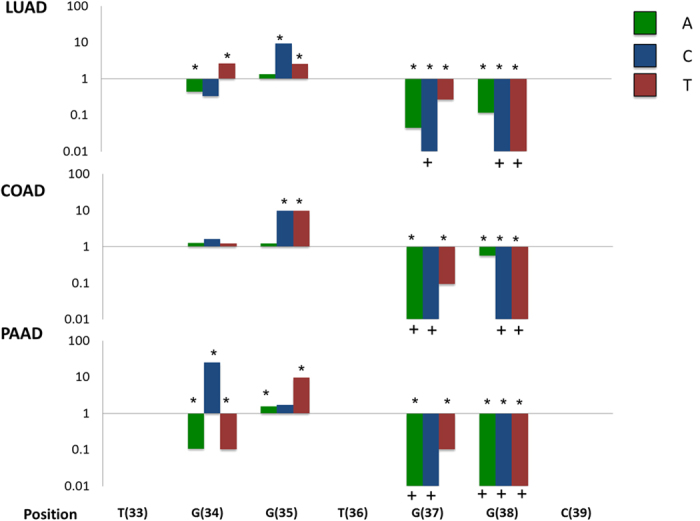
While some *KRAS* substitutions are over-represented relative mutational expectations across all tumor types, others are over-represented in only a single type of tumor. The values depicted are the observed relative frequency of each of the 12 possible *KRAS* driver substitutions, divided by the relative frequency expected under a model in which mutation alone determines the distribution of *KRAS* driver substitutions. The Y-axis is displayed in logarithmic scale. Values above and below one indicate an enrichment or depletion, respectively of a certain *KRAS* driver substitution, relative mutational expectations. Statistically significant under- or over-representation (according to a χ^2^ test) are marked by an asterisk (*P*-values are given in Table S4). Bars marked by a ‘+’ represent instances in which the observed number of *KRAS* driver substitution of this type was zero. Identities of substitutions are indicated by nucleic acid name and position (brackets) in the *KRAS* gene’s cDNA, at the bottom of the figure. LUAD = lung adenocarcinoma, COAD = colon adenocarcinoma, PAAD = pancreatic adenocarcinoma.

Next, we compared the observed relative frequencies of *KRAS* substitutions at codons 12 and 13 (Fig. 1), to those expected based on the mutational biases (Fig. 2). This allowed us to identify which of the *KRAS* driver substitutions within codons 12 and 13 are relatively over- and which are relatively under-represented, given mutational biases in the different tumor types (Fig. 3). Those mutations we identify as being relatively over-represented within a given tumor type are likely more favored by selection within that tumor type. At the same time, those mutations that are relatively under-represented are likely less favored within that type of tumor. It is important to note that both types of substitutions are likely subject to positive selection, as all *KRAS* codon 12 and 13 non-synonymous substitutions are known to be cancer drivers.

The c.35G > C and c.35G > T substitutions were over-represented relative to mutational expectations across all tumor types (although c.35G > C was only very slightly and not statistically significantly over-represented in PAAD (1.7 times the expected under mutational biases, Fig. 3, Table S4)). This likely indicates that these substitutions are relatively favored by natural selection across all types of tumors. In contrast we found that the c.34G > C, appears to be most favored only in PAAD, with a relative frequency ~24.5 times higher than expected from mutational biases. The same substitution is under-represented in LUAD, and only very slightly over-represented in COAD (Fig. 3). We also found that the c.34G > T substitution is approximately 2.6 times more frequent than expected from mutational biases in LUAD, but that the same substitution is strongly under-represented in PAAD, and appears about as frequently as expected from mutational biases in COAD. Of note, though under-represented across all tumor types, relative mutational expectations, c.38G > A appears to be less under-represented in colon compared to lung and pancreatic adenocarcinoma.

These results demonstrate that within tumors various *KRAS* driver substitutions are differently affected by natural selection, causing some driver substitutions to be over- or under-represented, relative expectations based on mutational biases. Some *KRAS* drivers seem to be more positively selected across tumor types, while others are strongly favored only in particular tumor types. Thus it appears that not all *KRAS* driver substitutions similarly affect fitness in all tumor types.

### Estimating the relative effect of mutational biases and natural selection on *KRAS* driver substitution distributions

Next, we sought to determine the relative influence of mutational biases and natural selection on the distribution of codon 12 and 13 *KRAS* driver substitutions. To do so, we assumed that in the absence of any mutational or selection biases each of the 12 possible driver substitutions would occur with equal frequencies, of approximately 8.3%. For each of the 12 possible substitutions types we then calculated the absolute fold change (see Methods) between its relative frequency under mutational biases (as calculated from data of NRSSs, Fig. 2) and the null hypothesis 8.3% frequency (equal distribution). This provided us with an estimate of the potential influence of mutational biases on the distribution of *KRAS* codon 12 and 13 driver substitutions. In order to estimate the influence of natural selection we calculated the absolute fold change between the observed frequency of each substitution (Fig. 1) and the frequency expected under mutational biases (Fig. 2).

In LUAD the average absolute fold change caused by mutation bias was 2.3 ± 1.9 whereas the average absolute fold change caused by natural selection was 7.3 ± 6.4 (a significant difference *P* = 0.002, according to a paired, one-tailed Mann-Whitney test). This indicates a larger influence of natural selection on the distribution of *KRAS* driver substitutions within LUAD. Similarly, in PAAD the average fold changes were 4.3 ± 3.8 and 13.1 ± 19.6 for mutation bias and natural selection, respectively. However, here the difference was only marginally significant (*P* = 0.06). In COAD, the relative contributions of mutation bias and natural selection were similar with 4.6  ±  4 and 5 ± 4.1 absolute fold change to mutation bias and natural selection, respectively (*P* = 0.6).

### Association between the presence of *KRAS* driver substitutions favored only in a specific tumor type and clinical outcomes, within that type of tumor

**Figure 4:**
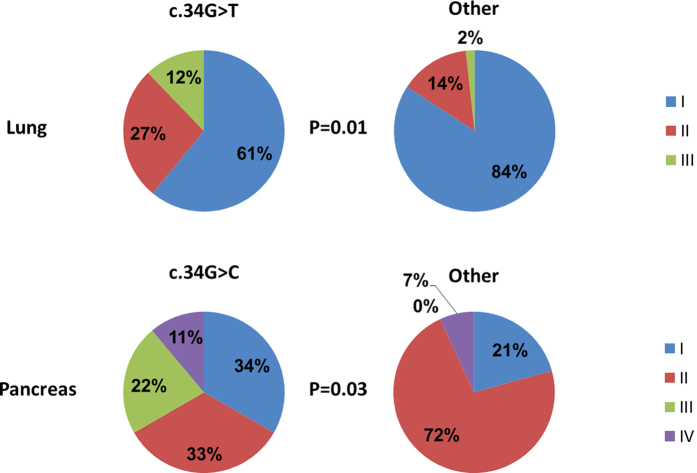
The presence of *KRAS* driver substitutions that we predicted as favored in only a single tumor type is associated with more advanced tumor stage, in the type of tumor in which they were predicted to be favored. This analysis was carried out on tumors from our local cohorts. Depicted are pie charts representing the stage distributions of tumors carrying the favored substitutions vs. tumors carrying any of the other possible *KRAS* driver substitutions.

We next wanted to examine whether the presence of *KRAS* substitutions we identify as being more favored by natural selection is associated clinical parameters. To this end we examined whether the presence of different *KRAS* substitution within tumors showed an association with higher tumor stage. For COAD, the local cohort analyzed contained only cases of the same stage, so we could not carry out this analysis. However, LUAD and PAAD tumors varied by stage. We found that the presence of the two favored substitutions that are shared by all tumor types (c.35G > C and c.35G > T) was not associated with stage within our local cohorts (*P* > 0.05, using a χ^2^ test). This could either stem from true lack of association, or from lack of power to detect association within our relatively small cohorts. At the same time within each tumor type the *KRAS* driver substitution that was identified as being favored only in that tumor type (c.34G > T for LUAD, and c.34G > C for PAAD) showed a significant association with tumor stage (*P* = 0.01 and 0.03, for LUAD and PAAD respectively, Fig. 4). Those substitutions that were not over-represented relative to mutational expectations were never found to significantly associate with tumor stage.

Our ability to examine association between clinical outcomes and the presence of certain *KRAS* driver substitutions was limited by the size of our dataset as well as by the fact that COAD patients were all of the same stage. We therefore next turned to examining whether the presence of *KRAS* substitutions we predict to be less or more favored within the various tumor types associated with clinical parameters according to external studies. c.34G > T, the substitution that was favored specifically in LUAD, was previously associated with considerably shorter PFS in lung carcinoma^13^,^14^, fitting with our finding that its presence is associated with higher tumor stage, in our local LUAD cohort. Additionally, the PAAD specifically favored substitution c.34G > C was also among the substitutions previously associated with worse prognosis in pancreatic adenocarcinoma^23^. Finally, the colon carcinoma specific (though not overrepresented) c.38G > A (p.G13D) substitution was previously found to associate with response to anti-EGFR antibodies and better overall survival (OS) and progression free survival (PFS)^16^, though the degree of response to anti-EGFR treatment is still under debate^19^. These and our findings suggest that the substitutions that are more favored in specific tissue represent a specific biologic entity with unique clinical phenotype.

### Smoking affects mutation and *KRAS* substitution frequency in lung but not in pancreatic cancer

**Figure 5:**
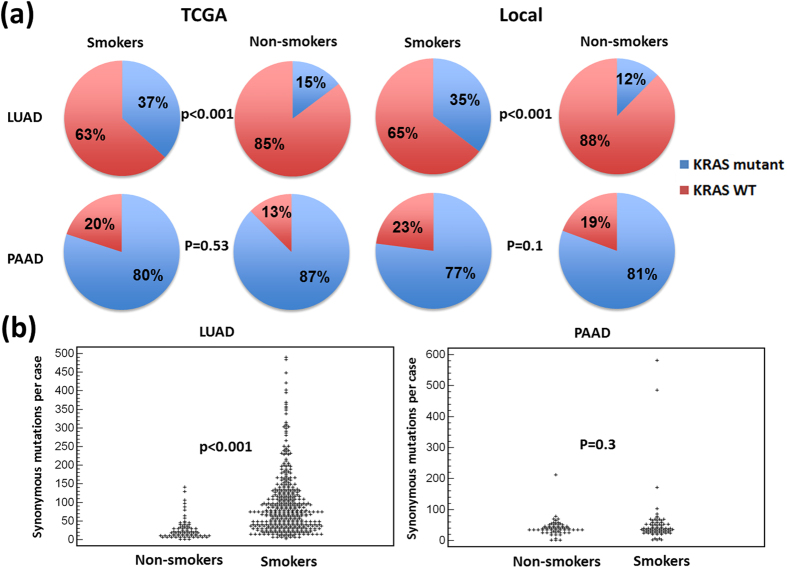
Smoking affects the frequency with which *KRAS* driver substitutions are found within tumors as well as overall mutation rates in LUAD but not in PAAD. (**A**) Depicted are pie charts representing the frequency with which *KRAS* is found within tumors of smokers and non-smokers for both PAAD and LUAD. (**B**) Depicted is the distribution of number of non-reoccurring synonymous substitutions found within tumors of smokers and non-smokers with PAAD and LUAD.

We found smoking to significantly increase the frequency of *KRAS* substitutions at codon 12 and 13 in LUAD (*P *= 0.0001, according to a χ^2^ test, Fig. 5A). However, no such difference was observed between smokers and non-smokers with PAAD (*P* = 0.53, Fig. 5A). Within LUAD, we also found that smokers tended on average to have a significantly higher number of non-reoccurring synonymous substitutions (NRSSs) within their tumors (*P* << 0.0001 according to a one-tailed Mann-Whitney test, Fig. 5B). In contrast no significant difference was found between the numbers of NRSSs found per tumor of smokers vs. non-smokers with PAAD (*P* = 0.3, Fig. 5B). Thus, it appears that smoking significantly increases overall mutation frequencies within LUAD tumors, but not within PAAD tumors.

Differences in the patterns of *KRAS* substitution and genome-wide mutation between smokers and non-smokers could not be studied meaningfully due to the relatively small numbers of non-smokers with LUAD and PAAD within our local cohort and the TCGA dataset.

## Discussion

Our analysis demonstrated that both mutational biases and differences in the manner in which natural selection affects various *KRAS* driver substitutions contribute to determining the distribution of *KRAS* driver substitutions within a tumor type. Why would the effects of natural selection be different on various *KRAS* driver substitutions? Previous reports have demonstrated that *KRAS* driver substitutions can be functionally quite different. For example, differences in the ability to induce transformation have been demonstrated between *KRAS* driver substitutions. *In-vitro* studies showed that the most transforming codon 12 substitutions are c.35G > T and c.34G > C^24^. Interestingly, our analysis predicted that c.35G > T is among the more favored substitutions in all three tumor types. c.34G > C is the substitution that is most over represented relative mutational expectations in PAAD. However, it is under represented in LUAD.

Different *KRAS* substitutions were also shown to vary in their downstream signaling effects. For example, c.34G > T and c.35G > T have been demonstrated to increase activation of ral signaling and decrease GF-dependent akt activation^13^. The over-representation of the c.34G > T (p.G12C) substitution within LUAD, may indicate the significance of ral signaling pathway in this malignancy^25^.

It is important to note that very different conclusions would be obtained had we only considered the frequency with which we observe different *KRAS* substitutions within tumors, without correcting for mutational biases. For example, it can be seen that the highly transforming c.34G > C (p.G12R) substitution appears quite infrequently within tumors, while the less potent c.35G > A (p.G12D) substitution appears much more frequently. Only after correcting for expected relative mutation rates, can we observe that c.34G > C is in fact highly over-represented within PAAD tumors, while c.35G > A appears at a relative frequency that fits mutational expectations. This highlights the contribution of mutational biases in determining the eventual frequencies with which different driver substitutions will appear within tumors.

We found that the presence of those *KRAS* driver substitutions that we predicted as favored by selection in only one tumor type correlated significantly with higher tumor stage, within that type of tumor. At the same time no such correlation was observed for the two substitutions that were predicted to be favored across all tumor types studied. While such a lack of correlation could be due to lack of statistical power, it could also represent and interesting trend. It could be that driver substitutions that are highly favored across tumor types may have roles that relate to the basic functions of the tumors. Could it be that such substitutions occur early on, and are therefore not predictive of stage? In contrast those substitutions that are favored only within a single type of tumor may have roles that are more specific. Of note, the association between the mutations and the clinical phenotype were found despite many other potentially confounding factors (differences in patient background clinical status, differences in the other somatic mutations within the tumors, etc.), thus indicating the strength of this association. Much more research on a much higher number of drivers will need to be carried out to determine whether indeed driver substitutions that are favored in specific tumor types tend to be better predictors of tumor stage, than driver substitutions that are favored across all tumor types.

Our results show that LUAD tumors from smokers are more than twice as likely as LUAD tumors form non-smokers to carry a *KRAS* driver substitution. At the same time PAAD tumors are as likely to carry *KRAS* driver substitutions, whether they come from smokers or from non-smokers. Mutational rates seem to follow a similar trend: they are significantly higher in smokers compared to non-smokers with LUAD, but more or less equal between smokers and non-smokers with PAAD.

The association between smoking and increased frequency of *KRAS* mutation in LUAD has been reported previously^26^,^27^,^28^,^29^,^30^ and indicates a clear link between smoking, mutagenesis and higher incidence of cancer. Although, it is not clear whether increased mutagenesis is the sole contributor to the increased incidence of LUAD in smokers.

A large number of studies have demonstrated that smoking increases the likelihood of PAAD^31^,^32^. Whether or not smoking increases the likelihood of PAAD tumors to carry *KRAS* and whether smoking increases overall mutation rates within PAAD tumors has been an area under debate^33^,^34^,^35^,^36^. Our results demonstrate no significant difference between smokers and non-smokers with PAAD in either the frequency of *KRAS* driver substitutions, or rates of mutation. Our results therefore indicate that smoking must increase the likelihood of PAAD by a mechanism different from increased mutagenesis. Possible mechanisms include reductions in the general fitness of the healthy tissue, making tumor cells more competitive^3^ or activation of tumor microenvironment either through increased inflammatory cytokines and reactive oxygen species^37^,^38^ or through recruitment and activation of tumor associated macrophages^39^. Additionally, previous reports have shown that nicotine metabolites can lead to dedifferentiation of acinar cells^40^ or cause epithelial to mesenchymal transition^41^. This in turn could affect the likelihood of developing cancer.

We could not examine whether smokers and non-smokers differed in their mutational biases and in their distributions of *KRAS* driver substitutions, because we had very scarce data of non-smokers with LUAD and PAAD. It will be highly interesting to understand whether for LUAD and PAAD, within each tumor type, there are differences in the distribution of *KRAS* substitutions between smokers and non-smokers. It would also be interesting to learn whether such differences are due entirely to differences in mutational biases, or whether natural selection sometimes favors different *KRAS* driver substitutions depending on smoking status. Towards this end it will be necessary to sequence more tumors originating from non-smokers with PAAD and LUAD.

Our approach can be extended to studying the effects of mutation and natural selection on determining the frequency distributions of other cancer driver substitutions within various types of tumors. In order to do so we will need to characterize on the one hand the relative frequencies with which various driver substitutions appear within tumors and on the other hand the mutational biases of those tumors. Here, we had a relatively simple case in which all possible driver substitutions involved a point mutation. Other driver substitutions within other genes can involve not only point mutations, but also sequence deletions, amplifications and chromosome rearrangements. In order to expand our approach to study such substitutions we will need to characterize not only the relative rates with which different types of point mutations occur, but also the relative rates with which other types of mutations occur.

To conclude, our results demonstrate that the frequency with which various *KRAS* driver substitutions appear within tumors is determined both by mutation and by natural selection. We show that within each tumor type selection favors certain *KRAS* driver substitutions over others. This differential effect of selection, together with mutational biases lead to a pattern by which various *KRAS* driver substitutions appear at very different frequencies within tumors. While some *KRAS* driver substitutions appear to be highly favored by natural selection across tumor types, some are highly favored only within specific types of tumors. Finally we show that the presence of those driver substitutions that are highly favored only in a specific type of tumor, within tumors of that specific type, correlates with tumor progression.

## Methods

### Patients

A local study group included 346 cases of localized lung adenocarcinoma that underwent surgical resection of the tumor between the years 2007–2011 and 47 cases of resected pancreatic adenocarcinoma. Additionally, 314 cases of metastatic colon carcinoma that underwent *KRAS* substitution testing as a part of their clinical evaluation for eligibility for anti-EGFR therapy were included in the study. Clinical, histopathological and survival data were collected for the available cases. All experimental protocols were approved by the Rambam Medical Center Ethics Committee and were carried out in accordance with the approved guidelines.

### DNA extraction and *KRAS* substitution analysis

DNA extraction from formalin-fixed-paraffin-embedded specimens was conducted as previously described^42^. Briefly, an area containing a high fraction of tumor cells was marked by a pathologist, microscopically dissected and DNA was extracted using the QuickExtract FFPE DNA Extraction kit (Epicentre, Madison, WI) according to manufacturer instructions. Following treatment with RNase A (Qiagen, Hilden, Germany), DNA was purified using the DNA Clean and Concentrator kit (Zymo Research, Orange, CA).

*KRAS* substitution analysis for the colon carcinoma samples was done by bidirectional Sanger sequencing, as previously described^43^. Briefly, DNA was PCR amplified using primers forward 5′-GGCCTGCTGAAAATGACTGAA-3′ and reverse 5′-GGTCCTGCACCAGTAATATGCA-3′. PCR amplification with Thermostart Master Mix (ABgene, NY, USA) was carried out by a hot start activation step of 95 °C for 15 min followed by 50 cycles of 95 °C for 30 s, 57 °C for 45 s, 72 °C for 1 min and 72 °C for 7 min for a final extension. Amplicons were subjected to direct (Sanger) DNA sequencing using the BigDye Terminator v1.1 Cycle Sequencing kit (Applied Biosystems, Foster city, CA) on an automated sequencer (ABI Prism 3130xl Genetic Analyzer; Applied Biosystems, Foster city, CA).

*KRAS* substitution analysis of the lung and pancreatic carcinoma samples was done by next generation sequencing (NGS) using the Ion Torrent Personal Genome Machine (PGM), as previously described^44^. For this purpose, *KRAS* primers were supplemented with Ion-Torrent adapters P1 and A, to allow binding to the Ion Sphere Particles (ISPs). Additionally, 96 different forward primers, each with a different barcode, were used for every genomic area amplified to allow the analysis of multiple samples in a single reaction. Amplicons were purified using the Qiagen PCR purification kit (Qiagen, Hilden, Germany) and were then sequenced using an Ion 314 chip and sequenced on the PGM for 65 cycles. Data from the PGM runs was initially processed using the Ion Torrent platform-specific pipeline software Torrent Suite v1.3.1 to generate sequence reads, trim adapter sequences, filter, and remove poor signal-profile reads. Generated sequence files were aligned to the genomic sequence of *KRAS* exon 2 and we determined mutation status manually, using the Integrative Genomic Viewer (IGV 2.3) free software^45^,^46^. The average coverage obtained for the local LUAD cases was 35970 ± 18857 (range 929–73679) and the average coverage for the local PAAD pancreas carcinoma cases was 8695 ± 5280 (range 1134–22112), allowing reliable identification of *KRAS* mutations in these cases.

Formalin fixation might induce sequence artifacts, most commonly deamination causing C:G > T:A transitions^47^. However, the frequency of such artifacts with the recent sequencing technologies is low^48^. Additionally, some sequencing errors, mostly indels in homopolymers, as well as coverage problems in GC rich areas, were associated with the use of the ion torrent technology^49^,^50^,^51^. Nevertheless, the lack of homopolymer area in the area we analyzed (codons 12&13 of *KRAS*), the fact that we looked for missense rather than indel mutations, the high sequencing coverage and the fact that we used technology-specific bioinformatics tools, built to address technology related biases make the possibility of misidentification of *KRAS* mutation in our samples very unlikely.

*KRAS* mutation analysis in the colon samples was performed prior to the introduction of NGS technology to the lab and was therefore analyzed by Sanger sequencing.

### TCGA data

Data of somatic substitutions in Colon Adenocarcinoma (COAD), Lung Adenocarcinoma (LUAD) and Pancreatic adenocarcinoma (PAAD) were downloaded on April 2015 from the Cancer Genome Atlas Project (TCGA) Data Portal^21^ (Table S1).

Smoking status of Lung adenocarcinoma and Pancreatic adenocarcinoma patients was extracted from clinical data downloaded on May 2015 (“tobacco smoking history” column). Patients marked as current or reformed smokers were considered as smokers. Only patients marked are lifelong non-smokers were considered as non-smokers.

We extracted known *KRAS* driver substitutions p.G12/c.34G, p.G12/c.35C, p.G13/c.37G, and p.G13/c.38G from the TCGA somatic data of COAD, LUAD and PAAD cancer types.

### Calculating mutation frequency based on TCGA data

Next, we wanted to calculate the expected relative frequency of each of the 12 possible *KRAS* codon 12 and 13 driver substitutions, in each tumor type, under a model in which mutational biases alone determine the distribution of *KRAS* driver substitutions. To do so, we extracted G > N synonymous substitutions from the TCGA somatic data. We considered only silent (synonymous) somatic substitutions that occurred in only one patient per cancer type.

One patient with Pancreatic Adenocarcinoma and four with Colon Adenocarcinoma were removed from consideration, as they had uncharacteristically high numbers of substitutions (the average number of substitutions per patient with PAAD and COAD is 52 and 100, respectively. We removed patients with over 1000 synonymous substitutions).

Within codons 12 and 13 of *KRAS* there are four guanine residues that can potentially harbor driver substitutions. Each of these G residues can change to A, C, or T giving a total of 12 potential driver substitutions. Mutation rates were shown to be influenced by context, meaning by which residues flank the mutated nucleotide. The contexts in which the four relevant *KRAS* G residues appear are tGg, gGt, tGg, and gGc (where the nucleotides flanking the G residue of interest are indicated in lowercase). We calculated the frequency with which we expect to observe mutations in G residues within these contexts. This allowed us to calculate the relative frequency with which we expect to observe each of the 12 possible substitutions under a model in which mutational biases alone determine the distribution of *KRAS* substitutions for the different tumor types.

To explain how we carried out our calculations, let us consider one of the 12 possible *KRAS* driver substitutions, c.34G > A. We can also represent this change as tGg to tAg, where the capital letters represent the actual nucleotide change and the lower case letters represent the flanking nucleotides. To calculate the expected mutation rate of this particular substation across the genome, within a specific cancer type, we counted how many single-occurrence synonymous substitutions from tGg to tAg occurred within the TCGA data of that tumor type, across the protein-coding genome. We then divided this number by the number of synonymous sites at which such a mutation could occur. To calculate the number of tGg to tAg synonymous sites, we first identified all G residues flanked by a 5′ T and a 3′ G within the human protein-coding gene sequences. For each gene, only the longest transcript was considered. It is important to note, that we looked at all tGg triplets, irrespective of whether they were in frame or not (meaning that tGg need not be a whole codon, but can rather belong to two adjacent codons). Each such G residue can potentially mutate to an A (as well as to the remaining two nucleotides). However, depending on the location of the G residue within the coding sequence, only some of the possible changes will be synonymous. The number of tGg > tAg synonymous sites was calculated as the number of tGg sites in which a tGg to tAg change would maintain the identity of the protein-coding sequence.

In this manner we calculated the expected mutation rate (r) of each of the 12 possible *KRAS* substitutions. Next, we calculated the expected relative rate (rr) of each substitution relative other substitutions, under a model in which mutational biases determine *KRAS* substitution distributions, as presented in equation number 1:


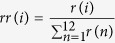


(i goes from 1 to 12 representing all 12 possible *KRAS* codon 12 and 13 driver substitutions).

In order to calculate significance using the χ^2^ test, one needs to compare numbers and not relative rates. The expected number of *KRAS* substitutions of each type, under a model in which mutational biases determine *KRAS* driver substitution distribution was calculated as presented in equation number 2:


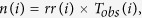


where, n(i) is the number of times substitution i is expected to appear within our data, rr(i) is the expected relative rate of the i substitution, and T_obs_ is the total number of observed *KRAS* substitutions in the considered tumor type.

Table S3 summarizes all calculation steps for the three studied cancer types.

### Estimating the relative influence of mutation and natural selection on determining *KRAS* driver substitution distributions

“Absolute fold change” was defined as the ratio between the higher and lower values compared. The expected frequency of each of the 12 possible alterations in the absence of any mutation or selection bias would be 8.33% (100%/12). To determine the effect of mutational biases we calculated the absolute fold change between the mutational expectation frequencies estimated through the analysis of the non-reoccurring synonymous substitutions (NRSSs), and the 8.33% frequency expected under no bias. The effect of natural selection was estimated by calculating the absolute fold change between the mutational expectation frequency of each alteration (based on NRSSs, depicted in Fig. 2) and the observed frequency of each *KRAS* substitution (Fig. 1). *KRAS* substitutions that were not present were assigned a dummy value of 0.5. The results are summarized in Table S5.

## Additional Information

**How to cite this article**: Ostrow, S. L. *et al.* Variation in *KRAS* driver substitution distributions between tumor types is determined by both mutation and natural selection. *Sci. Rep.* **6**, 21927; doi: 10.1038/srep21927 (2016).

## Supplementary Material

Supplementary Information

## Figures and Tables

**Table 1:** The fraction of patients carrying a *KRAS* codon 12 or 13 driver substitution varies between tumor types.

	TCGA		Local patient cohorts	
	*KRAS* positive	*KRAS* negative	*KRAS* positive	*KRAS* negative
Lung Adenocarcinoma (LUAD)	164 (30%)	382 (70%)	99 (29%)	247 (71%)
Colon Adenocarcinoma (COAD)	72 (27%)	193 (73%)	125 (40%)	189 (60%)
Pancreatic Adenocarcinoma (PAAD)	128 (75%)	42 (25%)	38 (81%)	9 (19%)
