# Supplementary material for: Variation in KRAS driver substitution distributions between tumor types is determined by both mutation and natural selection
Source: Sci Rep. 2016 Feb 23;6:21927. doi: 10.1038/srep21927 (PMC4763303; doi:10.1038/srep21927)
Supplement: Supplementary Information [file srep21927-s1.doc]

**Variations in *KRAS* substitutions between and within tumors are contributed by both mutation and natural selection**

Sheli L. Ostrow, Einav Simon, Elad Prinz, Tova Bick, Talia Shentzer, Sima S. Nagawkar, Edmond Sabo, Ofer Ben-Izhak, Ruth Hershberg, Dov Hershkovitz

Table S1 Summary of TCGA datasets

|  | Number of patients | Number of known *KRAS* substitutions | NRSS^a^ |
| --- | --- | --- | --- |
| LUAD | 546 | 167 | 45616 |
| COAD | 265 | 73 | 22946 |
| PAAD | 170 | 132 | 6201 |

^a^# of Non-reoccurring synonymous substitutions

Table S2 – Observed KRAS substitution distribution

| KRAS mutation | | Lung adenocarcinoma | | | | Colon adenocarcinoma | | | | Pancreatic adenocarcinoma | | | |
| --- | --- | --- | --- | --- | --- | --- | --- | --- | --- | --- | --- | --- | --- |
| AA sub^a^ | Base sub^a^ | TCGA | | Local | | TCGA | | Local | | TCGA | | Local | |
|  |  | Subs^a^ count | Subs^a^ percent | Subs^a^ count | Subs^a^ percent | Subs^a^ count | Subs^a^ percent | Subs^a^ count | Subs^a^ percent | Subs^a^ count | Subs^a^ percent | Subs^a^ count | Subs^a^ percent |
| p.G12S | c.34G>A | 4 | 2.40 | 5 | 5.00 | 5 | 6.85 | 9 | 7.20 | 1 | 0.76 | 0 | 0.00 |
| p.G12R | c.34G>C | 1 | 0.60 | 2 | 2.00 | 3 | 4.11 | 0 | 0.00 | 27 | 20.45 | 9 | 23.68 |
| p.G12C | c.34G>T | 74 | 44.31 | 43 | 43.00 | 4 | 5.48 | 9 | 7.20 | 1 | 0.76 | 0 | 0.00 |
| p.G12D | c.35G>A | 21 | 12.57 | 22 | 22.00 | 22 | 30.14 | 51 | 40.80 | 61 | 46.21 | 11 | 28.95 |
| p.G12A | c.35G>C | 17 | 10.18 | 9 | 9.00 | 5 | 6.85 | 6 | 4.80 | 1 | 0.76 | 1 | 2.63 |
| p.G12V | c.35G>T | 41 | 24.55 | 13 | 13.00 | 15 | 20.55 | 29 | 23.20 | 40 | 30.30 | 17 | 44.74 |
| p.G13S | c.37G>A | 0 | 0.00 | 1 | 1.00 | 0 | 0.00 | 0 | 0.00 | 0 | 0.00 | 0 | 0.00 |
| p.G13R | c.37G>C | 0 | 0.00 | 0 | 0.00 | 0 | 0.00 | 0 | 0.00 | 0 | 0.00 | 0 | 0.00 |
| p.G13C | c.37G>T | 7 | 4.19 | 4 | 4.00 | 1 | 1.37 | 0 | 0.00 | 1 | 0.76 | 0 | 0.00 |
| p.G13D | c.38G>A | 2 | 1.20 | 1 | 1.00 | 18 | 24.66 | 21 | 16.80 | 0 | 0.00 | 0 | 0.00 |
| p.G13A | c.38G>C | 0 | 0.00 | 0 | 0.00 | 0 | 0.00 | 0 | 0.00 | 0 | 0.00 | 0 | 0.00 |
| p.G13V | c.38G>T | 0 | 0.00 | 0 | 0.00 | 0 | 0.00 | 0 | 0.00 | 0 | 0.00 | 0 | 0.00 |

^a^ Substitution

Table S3 – Calculating expected substitution distribution under mutational biases

| KRAS mutation | | Mutation and context | Syn^b^  sites | Lung adenocarcinoma | | | | Colon adenocarcinoma | | | | Pancreatic adenocarcinoma | | | |
| --- | --- | --- | --- | --- | --- | --- | --- | --- | --- | --- | --- | --- | --- | --- | --- |
| AA sub^a^ | Base sub^a^ |  |  | Syn^b^ sub^a^ | Syn^b^ rate | Expected count | Expected percent | Syn^b^ sub^a^ | Syn^b^ rate | Expected count | Expected percent | Syn^b^ sub^a^ | Syn^b^ rate | Expected count | Expected percent |
| p.G12S | c.34G>A | TAG | 382890 | 1561 | 0.0041 | 13.1056 | 7.8477 | 342 | 0.0009 | 4.1541 | 5.6906 | 92 | 0.0002 | 7.3452 | 5.5645 |
| p.G12R | c.34G>C | TCG | 322143 | 570 | 0.0018 | 5.6880 | 3.4060 | 49 | 0.0002 | 0.7074 | 0.9691 | 12 | 0.0000 | 1.1387 | 0.8627 |
| p.G12C | c.34G>T | TTG | 322143 | 2818 | 0.0087 | 28.1204 | 16.8386 | 278 | 0.0009 | 4.0135 | 5.4979 | 81 | 0.0003 | 7.6864 | 5.8231 |
| p.G12D | c.35G>A | GAT | 51519 | 331 | 0.0064 | 20.6533 | 12.3673 | 249 | 0.0048 | 22.4781 | 30.7919 | 62 | 0.0012 | 36.7886 | 27.8701 |
| p.G12A | c.35G>C | GCT | 32789 | 18 | 0.0005 | 1.7647 | 1.0567 | 3 | 0.0001 | 0.4255 | 0.5829 | 1 | 0.0000 | 0.9323 | 0.7063 |
| p.G12V | c.35G>T | GTT | 32789 | 135 | 0.0041 | 13.2353 | 7.9254 | 12 | 0.0004 | 1.7021 | 2.3316 | 5 | 0.0002 | 4.6615 | 3.5315 |
| p.G13S | c.37G>A | TAG | 382890 | 1561 | 0.0041 | 13.1056 | 7.8477 | 342 | 0.0009 | 4.1541 | 5.6906 | 92 | 0.0002 | 7.3452 | 5.5645 |
| p.G13R | c.37G>C | TCG | 322143 | 570 | 0.0018 | 5.6880 | 3.4060 | 49 | 0.0002 | 0.7074 | 0.9691 | 12 | 0.0000 | 1.1387 | 0.8627 |
| p.G13C | c.37G>T | TTG | 322143 | 2818 | 0.0087 | 28.1204 | 16.8386 | 278 | 0.0009 | 4.0135 | 5.4979 | 81 | 0.0003 | 7.6864 | 5.8231 |
| p.G13D | c.38G>A | GAC | 140843 | 600 | 0.0043 | 13.6945 | 8.2003 | 784 | 0.0056 | 25.8885 | 35.4638 | 220 | 0.0016 | 47.7503 | 36.1744 |
| p.G13A | c.38G>C | GCC | 102683 | 138 | 0.0013 | 4.3203 | 2.5870 | 32 | 0.0003 | 1.4494 | 1.9854 | 14 | 0.0001 | 4.1679 | 3.1575 |
| p.G13V | c.38G>T | GTC | 102683 | 623 | 0.0061 | 19.5038 | 11.6789 | 73 | 0.0007 | 3.3064 | 4.5293 | 18 | 0.0002 | 5.3587 | 4.0596 |

^a^ Substitution

^b^ Synonymous

Table S4. Significance of over or under-representation of each of the 12 driver substitutions relative mutational expectations

|  | LUAD | COAD | PAAD |
| --- | --- | --- | --- |
| c.34G>A | 0.02418 | 0.574352 | 0.007032 |
| c.34G>C | 0.074996 | 0.623777 | 1.10E-09 |
| c.34G>T | 1.04E-11 | 0.655851 | 0.005428 |
| c.35G>A | 0.216466 | 0.201052 | 0.005121 |
| c.35G>C | 6.77E-06 | 0.003589 | 0.652776 |
| c.35G>T | 4.14E-05 | 9.98E-10 | 5.74E-13 |
| c.37G>A | 9.24E-06 | 0.000466 | 0.00135 |
| c.37G>C | 0.001775 | 0.153288 | 0.211583 |
| c.37G>T | 1.36E-06 | 0.003102 | 0.005428 |
| c.38G>A | 8.67E-05 | 0.000442 | 7.50E-19 |
| c.38G>C | 0.006533 | 0.04056 | 0.016361 |
| c.38G>T | 3.46E-09 | 0.001852 | 0.006386 |

Table S5. The relative influence of mutational biases and natural selection on the distribution of codon 12 and 13 KRAS driver substitutions

|  | | LUAD | | | | COAD | | | | PAAD | | | |
| --- | --- | --- | --- | --- | --- | --- | --- | --- | --- | --- | --- | --- | --- |
|  |  |  |  | Absolute fold change | |  |  | Absolute fold change | |  |  | Absolute fold change | |
| AA sub | Base sub | Mut exp %^a^ | Obs %^b^ | Mutation bias | Natural selection | Mut exp %^a^ | Obs %^b^ | Mutation bias | Natural selection | Mut exp %^a^ | Obs %^b^ | Mutation bias | Natural selection |
| p.G12S | c.34G>A | 7.8 | 2.4 | 1.1 | 3.3 | 5.7 | 6.9 | 1.5 | 1.2 | 5.6 | 0.8 | 1.5 | 7.3 |
| p.G12R | c.34G>C | 3.4 | 0.6 | 2.4 | 5.7 | 1.0 | 4.1 | 8.6 | 4.2 | 0.9 | 20.5 | 9.7 | 23.7 |
| p.G12C | c.34G>T | 16.8 | 44.3 | 2.0 | 2.6 | 5.5 | 5.5 | 1.5 | 1.0 | 5.8 | 0.8 | 1.4 | 7.7 |
| p.G12D | c.35G>A | 12.4 | 12.6 | 1.5 | 1.0 | 30.8 | 30.1 | 3.7 | 1.0 | 27.9 | 46.2 | 3.3 | 1.7 |
| p.G12A | c.35G>C | 1.1 | 10.2 | 7.9 | 9.6 | 0.6 | 6.9 | 14.3 | 11.8 | 0.7 | 0.8 | 11.8 | 1.1 |
| p.G12V | c.35G>T | 7.9 | 24.6 | 1.1 | 3.1 | 2.3 | 20.6 | 3.6 | 8.8 | 3.5 | 30.3 | 2.4 | 8.6 |
| p.G13S | c.37G>A | 7.8 | 0.5 | 1.1 | 15.7 | 5.7 | 0.5 | 1.5 | 11.4 | 5.6 | 0.5 | 1.5 | 11.1 |
| p.G13R | c.37G>C | 3.4 | 0.5 | 2.4 | 6.8 | 1.0 | 0.5 | 8.6 | 1.9 | 0.9 | 0.5 | 9.7 | 1.7 |
| p.G13C | c.37G>T | 16.8 | 4.2 | 2.0 | 4.0 | 5.5 | 1.4 | 1.5 | 4.0 | 5.8 | 0.8 | 1.4 | 7.7 |
| p.G13D | c.38G>A | 8.2 | 1.2 | 1.0 | 6.8 | 35.5 | 24.7 | 4.3 | 1.4 | 36.2 | 0.5 | 4.3 | 72.3 |
| p.G13A | c.38G>C | 2.6 | 0.5 | 3.2 | 5.2 | 2.0 | 0.5 | 4.2 | 4.0 | 3.2 | 0.5 | 2.6 | 6.3 |
| p.G13V | c.38G>T | 11.7 | 0.5 | 1.4 | 23.4 | 4.5 | 0.5 | 1.8 | 9.1 | 4.1 | 0.5 | 2.1 | 8.1 |

^a^The percentage with which this substitution would be expected to occur under a model in which the distribution of KRAS driver substitutions was determined solely by mutational biases (estimated based on data of NRSSs)

^b^The observed frequency with which each KRAS driver substitution appeared.
